# Effect of volatile and total intravenous anesthesia on syndecan-1 shedding after minimally invasive gastrectomy: a randomized trial

**DOI:** 10.1038/s41598-021-81012-1

**Published:** 2021-01-15

**Authors:** Na Young Kim, Ki Jun Kim, Ki-Young Lee, Hye Jung Shin, Jaein Cho, Da Jeong Nam, So Yeon Kim

**Affiliations:** 1 Department of Anesthesiology and Pain Medicine and Anesthesia and Pain Research Institute, Yonsei University College of Medicine, 50-1 Yonsei-ro, Seodaemun-gu, Seoul, 03722 Republic of Korea; 2 Biostatistics Collaboration Unit, Yonsei University College of Medicine, Seoul, Republic of Korea; 3 Department of Anaesthesiology and Pain Medicine, National Health Insurance Service Ilsan Hospital, Goyang, Republic of Korea

**Keywords:** Biomarkers, Gastroenterology

## Abstract

This study aimed to compare the effects of volatile anesthesia and total intravenous anesthesia (TIVA) on syndecan-1 shedding in patients with gastric cancer undergoing minimally invasive gastrectomy. Patients were randomly assigned to either the Volatile (n = 68) or the TIVA (n = 68) group. Anesthesia was maintained with sevoflurane/remifentanil or propofol/remifentanil in the Volatile and TIVA groups, respectively. Serum syndecan-1 was evaluated at pre-operation, end of operation, and postoperative day (POD) 1. Inflammatory markers including white blood cell (WBC) count, neutrophil-to-lymphocyte ratio (NLR), and C-reactive protein (CRP), were also measured at pre-operation, end of operation, and POD 1, 2, 3, and 5. The TIVA group showed significantly lower levels of syndecan-1 at the end of the operation compared to the Volatile group; however, no difference was seen between the groups at POD 1. The WBC count and NLR were significantly lower in the TIVA group at the end of the operation than the Volatile group, but there were no differences between the groups at POD 1, 2, 3, and 5. CRP levels were similar between the groups at all time points. In conclusion, despite TIVA being superior to volatile anesthesia in protecting endothelial glycocalyx during the operation, both did not prevent postoperative syndecan-1 shedding after gastrectomy.

**Clinical trial registration number**: NCT04183296 (ClinicalTrial.gov, 03/12/2019).

## Introduction

The endothelial glycocalyx (EG) is a gel-like layer that coats the luminal surface of the vascular endothelium, with thicknesses between 0.2 and 2 μm depending on the type of vasculature^1–3^. The EG is degraded under pathological conditions, such as ischemia–reperfusion (I/R) injury, oxidative stress, and inflammation^1–3^. Proteoglycans are the most important backbone molecule of the EG, and heparan sulfate proteoglycans represent about 50–90% of the proteoglycans^1,2^. Syndecans are a family of heparan sulfate proteoglycan, and syndecan-1 has been widely studied as a marker of EG breakdown in various conditions, including in surgical patients^4–13^.

Since no pharmacologic agents for the restoration of the EG are clinically available, strategies to prevent EG degradation in surgical patients are needed^3^. There have been experimental studies on the protective effect of sevoflurane against EG degradation from I/R injury^14–16^. Sevoflurane was superior to propofol in protecting the EG from I/R injury in a porcine model^16^. In contrast to experimental results, sevoflurane did not show a better protective effect on the EG than propofol in clinical studies of lung resection surgery and knee-ligament surgery^9,10^.

Minimally invasive laparoscopic surgery causes less surgical trauma compared to open surgery, which leads to less stress and attenuated inflammatory response^17,18^. In minimally invasive abdominal surgery, pneumoperitoneum is essential for adequate visualization and working space. However, abdomen insufflation causes a reduction in splanchnic blood flow, which causes organ ischemia followed by reperfusion injury upon deflation of the abdomen, resulting in oxidative stress^19^. Although I/R injury and oxidative stress are highly related to EG disruption, no study has evaluated the perioperative EG changes in minimally invasive abdominal surgery with pneumoperitoneum, nor the effect of different general anesthetics on these changes. Hence, this randomized controlled trial aimed to compare the effect of volatile anesthesia with sevoflurane/remifentanil and of total intravenous anesthesia (TIVA) with propofol/remifentanil on syndecan-1 shedding in patients with gastric cancer undergoing laparoscopic or robotic gastrectomy.

## Results

### Demographic and intraoperative characteristics

**Figure 1:**
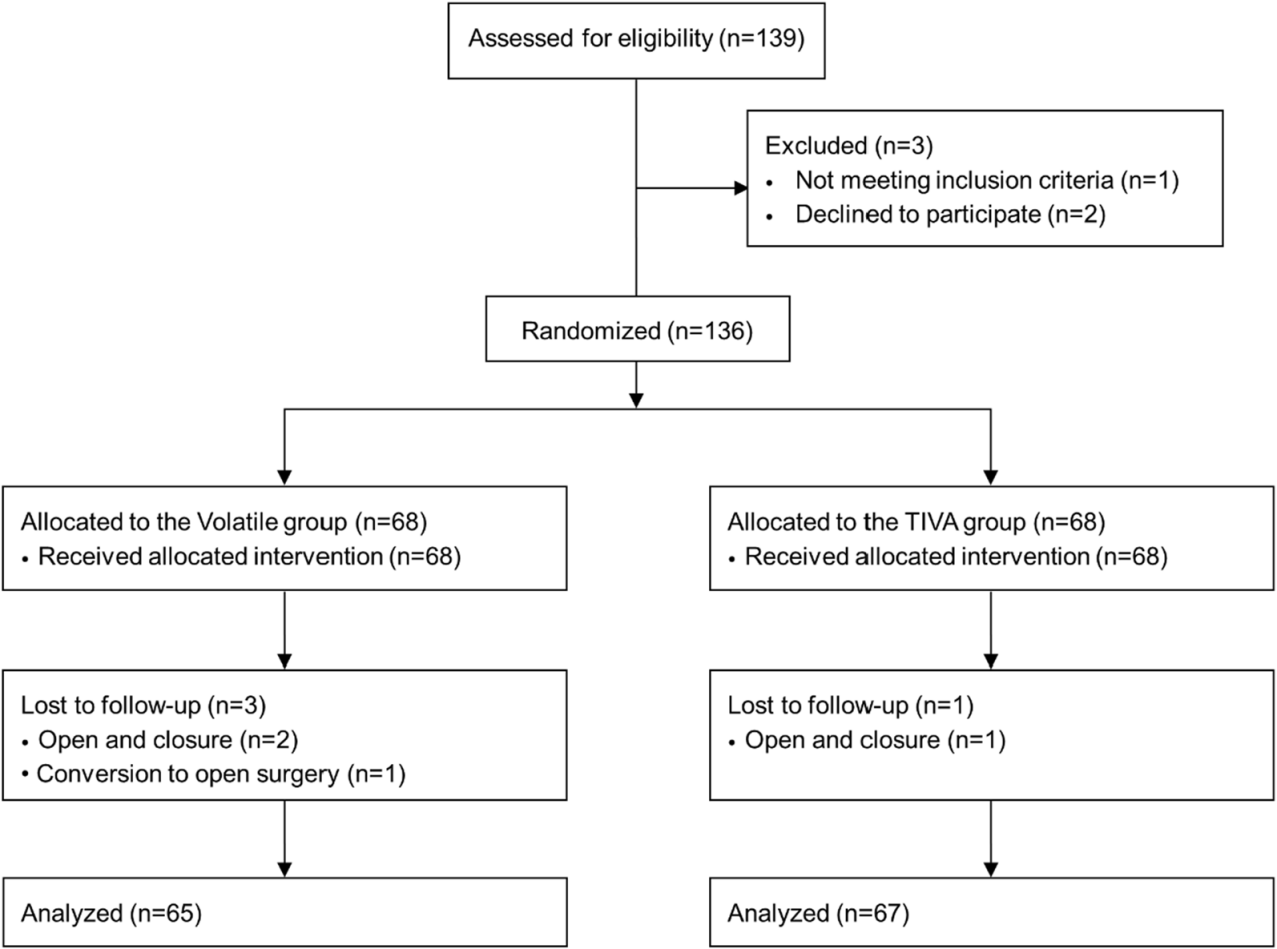
Consort flow diagram of the study.

**Figure 2:**
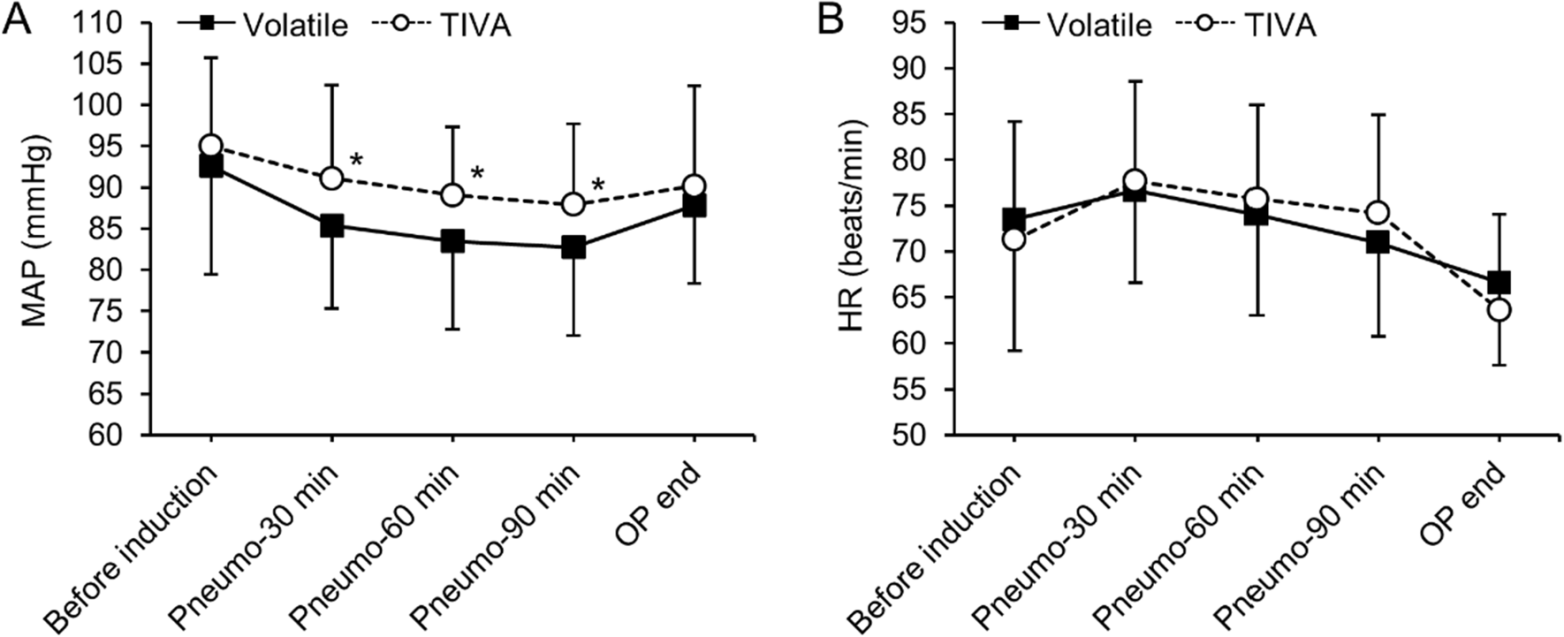
Intraoperative changes in (**A**) mean arterial pressure (MAP) and (**B**) heart rate (HR). Pneumo, pneumoperitoneum; OP, operation; TIVA, total intravenous anesthesia. Values are expressed as mean ± SD. *Bonferroni-corrected *P* < 0.05 versus Volatile group.

Of the 139 patients assessed for eligibility, 136 patients were randomly assigned into the two groups, and 132 of those patients completed the study (97%). Four patients were excluded from the final analysis (three patients from the Volatile group and one from the TIVA group) because of open and closure or conversion to open surgery (Fig. 1). The patients’ characteristics and intraoperative variables are shown in Table 1. The administered dose of remifentanil was significantly higher in the TIVA group (*P* < 0.001), whereas significantly higher doses of ephedrine and phenylephrine were administered to the Volatile group (both *P* < 0.001). No differences were observed between the two groups in other variables. Intraoperative MAP was significantly higher in the TIVA group at 30, 60, and 90 min after pneumoperitoneum compared to the Volatile group (Bonferroni corrected *P* = 0.012, 0.016, and 0.034, respectively; Fig. 2A). No statistical differences in heart rate were found between the two groups (Fig. 2B). Arterial partial pressures of oxygen and carbon dioxide at 90 min after pneumoperitoneum showed no significant differences between the Volatile and TIVA groups (202 ± 31 vs 206 ± 31 mmHg, *P* = 0.642 and 39 ± 4 vs 39 ± 3 mmHg, *P* = 0.977, respectively).

**Table 1:** Patients’ characteristics and intra-operative variables

Variable	Volatile group (n = 65)	TIVA group (n = 67)	*P* value
Age (year)	64.6 ± 10.4	61.5 ± 9.0	0.068
Male sex	37 (57%)	29 (43%)	0.117
Body mass index (kg/m^2^)	23.2 ± 2.9	24.0 ± 2.8	0.108
**ASA physical status**			0.688
I/II/III	9 (14%)/39 (60%)/17 (26%)	8 (12%)/45 (67%)/14 (21%)	
**Co-morbidities**			
Hypertension	27 (42%)	18 (27%)	0.075
Diabetes mellitus	13 (20%)	8 (12%)	0.206
**Type of operation**			0.834
Robotic/Laparoscopic	26 (40%)/39 (60%)	28 (42%)/39 (58%)	
**Type of gastrectomy**			0.420
Subtotal/Total/Proximal subtotal	51 (79%)/12 (18%)/2 (3%)	58 (87%)/7 (10%)/2 (3%)	
**Type of reconstruction**			0.091
Billoth I	36 (55%)	40 (60%)	
Billoth II	8 (12%)	15 (22%)	
Double tract	3 (5%)	4 (6%)	
Roux-en-Y	18 (28%)	8 (12%)	
**Extent of lymph node dissection**			0.383
D1/D2	43 (66%)/22 (34%)	49 (73%)/18 (27%)	
**TNM stage**			0.788
I/II/III/IV	41 (63%)/14 (21%)/7 (11%)/3 (5%)	47 (70%)/12 (18%)/4 (6%)/4 (6%)	
**Intra-operative variables**			
Pneumoperitoneum time (min)	151 ± 42	149 ± 47	0.769
Operation time (min)	183 ± 43	182 ± 49	0.923
Anesthesia time (min)	214 ± 44	213 ± 48	0.938
Crystalloid intake (mL)	1254 ± 413	1331 ± 436	0.300
Colloid intake (mL)	368 ± 284	321 ± 253	0.319
Blood loss (mL)	69 ± 65	68 ± 90	0.936
Urine output (mL)	191 ± 98	181 ± 112	0.590
Remifentanil dose (μg)	940 ± 330	1294 ± 464	< 0.001
Ephedrine dose (mg)	6.8 ± 5.7	3.4 ± 4.9	< 0.001
Phenylephrine dose (μg)	1522 ± 2026	411 ± 1008	< 0.001

Values are presented as mean ± SD or number of patients (percentage). TIVA: total intravenous anesthesia; ASA: American Society of Anesthesiologists; TNM: tumor-node-metastasis.

### Syndecan-1

**Figure 3:**
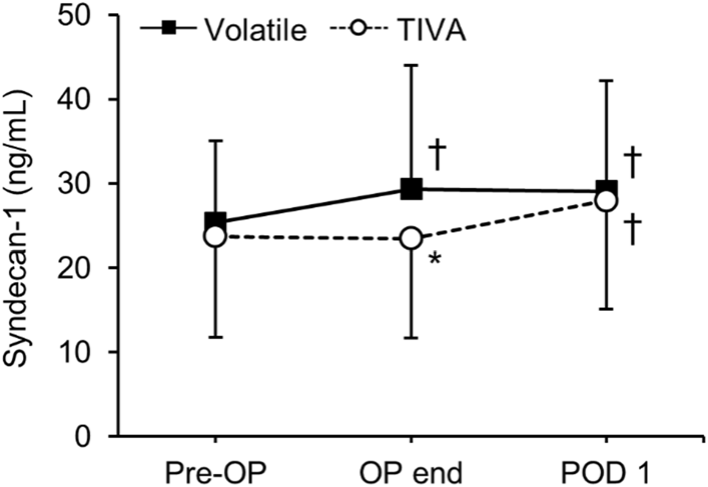
Perioperative changes in serum syndecan-1. OP, operation; POD, postoperative day; TIVA, total intravenous anesthesia. Values are expressed as mean ± SD. *Bonferroni-corrected *P* < 0.05 versus Volatile group. ^†^Bonferroni-corrected *P* < 0.05 versus Pre-OP in each group.

The serum concentration of syndecan-1 was significantly increased at the end of the operation compared to preoperative values, and this increase was maintained until POD 1 in the Volatile group. However, in the TIVA group, syndecan-1 was significantly elevated at POD 1 but not at the end of the operation. Therefore, the TIVA group showed significantly lower levels of syndecan-1 at the end of the operation compared to the Volatile group (23.4 ± 11.7 vs 29.3 ± 14.7 ng/mL; Bonferroni corrected *P* = 0.021), but no difference existed between the groups at POD 1 (29.1 ± 13.1 ng/mL in the Volatile group vs 28.0 ± 12.9 ng/mL in the TIVA group; Fig. 3). Even after adjusting for confounding variables (MAP, dose of ephedrine, and dose of phenylephrine) which may contribute to organ ischemia, the syndecan-1 level was significantly lower in the TIVA group at the end of the operation compared to the Volatile group (Bonferroni corrected *P* = 0.042, data not shown), while there was no difference between the groups at POD1. When compared to preoperative values, 19 patients (44%) in the Volatile group and 24 patients (56%) in the TIVA group presented an elevation of syndecan-1 of more than 30% at POD 1 (*P* = 0.149, data not shown).

### WBC count, NLR, and CRP

**Figure 4:**
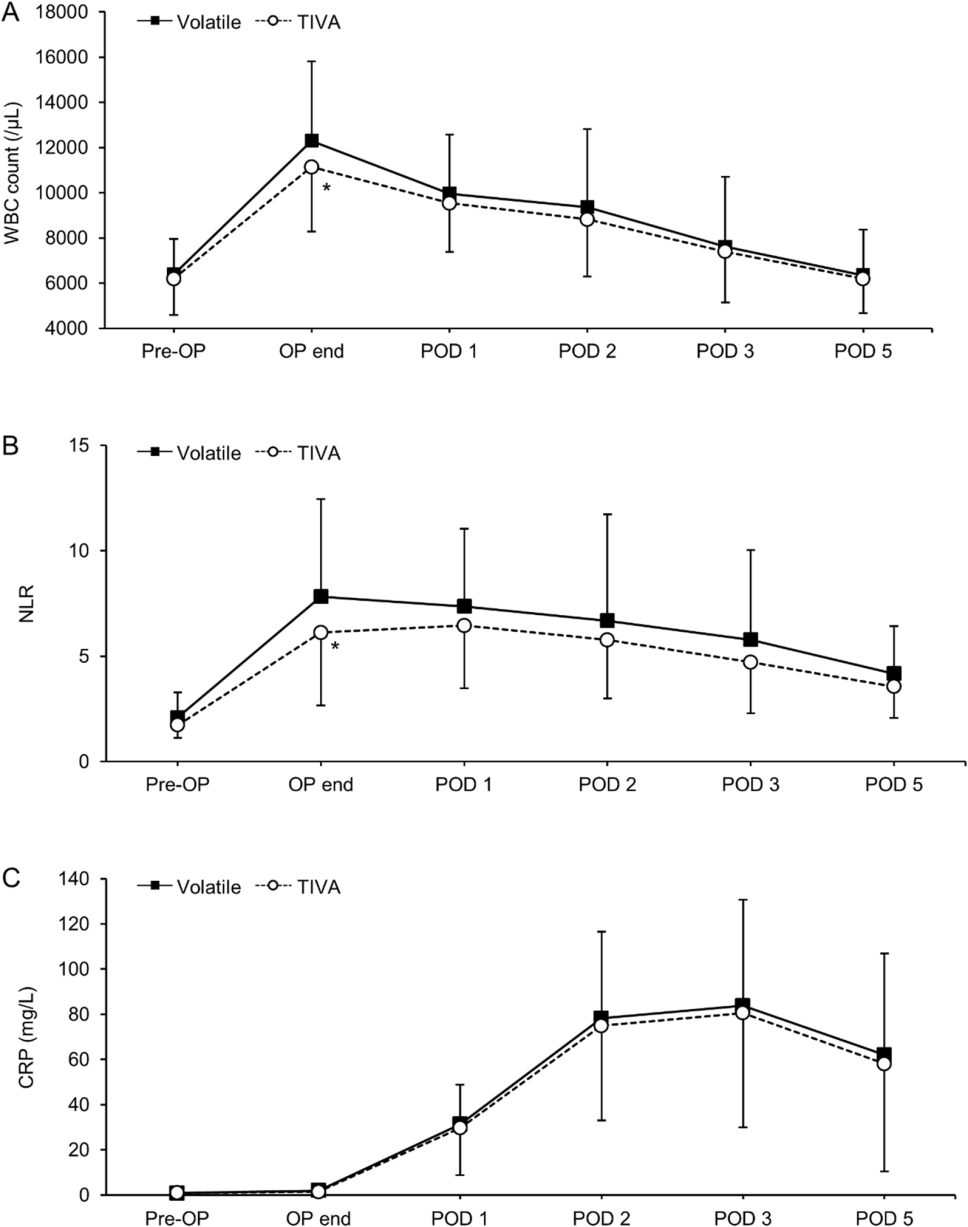
Perioperative changes in (**A**) white blood cell (WBC) counts, (**B**) neutrophil/lymphocyte ratio (NLR), and (**C**) C-reactive protein (CRP). OP, operation; POD, postoperative day; TIVA, total intravenous anesthesia. Values are expressed as mean ± SD. *Bonferroni-corrected *P* < 0.05 versus Volatile group.

The WBC count and NLR were significantly lower in the TIVA group at the end of the operation than in the Volatile group (Bonferroni corrected *P* = 0.049 and 0.013, respectively), while no differences were found between the groups at POD 1, 2, 3, and 5 (Fig. 4A,B). In addition, there was no difference in the CRP levels between the groups at any time point (Fig. 4C).

### Postoperative consumption of analgesics and hospital stay

**Figure 5:**
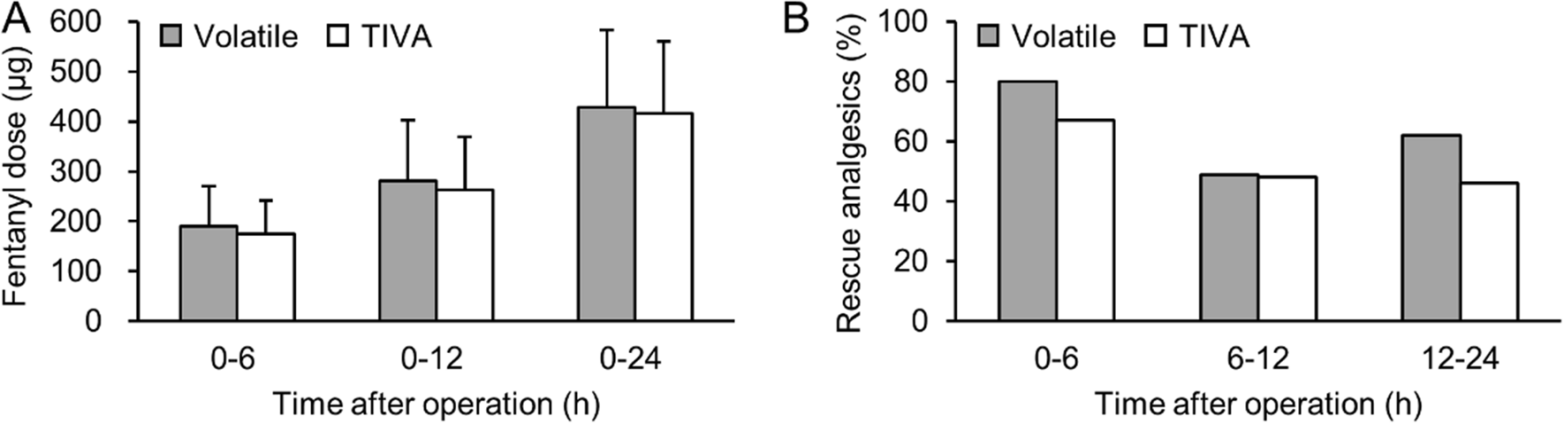
(**A**) Cumulative fentanyl dose administered via intravenous patient-controlled analgesia, and (**B**) use of rescue analgesics during the first 24 h after operation. TIVA, total intravenous anesthesia. Values are expressed as mean ± SD or percentage.

The cumulative doses of fentanyl at postoperative 6, 12, and 24 h were similar in both groups (Fig. 5A). Likewise, no significant differences were observed in the number of patients who required rescue analgesics between the two groups (Fig. 5B). The postoperative hospital stay was of 6.5 ± 3.5 days in the Volatile group and 6.0 ± 2.2 days in the TIVA (*P* = 0.352).

## Discussion

This randomized controlled study was the first to explore the perioperative changes of syndecan-1 and the effects of two commonly used general anesthetics on these changes in minimally invasive abdominal surgery with pneumoperitoneum. Our study showed that the serum concentration of syndecan-1 was significantly increased at POD 1 compared to preoperative levels, and that the increase was comparable between patients under volatile anesthesia with sevoflurane/remifentanil and those under TIVA with propofol/remifentanil. Our results further confirmed previous findings indicating that sevoflurane and propofol have similar effects regarding perioperative EG degradation^9,10^.

The EG layer is a fine structure that lines the endoluminal surface of the endothelium and acts as a regulator of endothelial function^1–3^. It is a labile structure and can be easily destroyed by a variety of enzymes and reactive oxygen species under inflammatory conditions^1–3^. Acute degradation of the EG has been demonstrated in patients after cardiac surgery, sepsis, and major trauma, and elevation of EG markers in blood is associated with poor outcomes in these patients^3,6,8^. Although previous studies have shown EG shedding in patients after major abdominal surgery^5,7^, literature on EG degradation after minimally invasive abdominal surgery was scarce. Minimally invasive abdominal surgery implies less inflammatory response compared to open surgical techniques^17,18^, from which it could be inferred that minimally invasive abdominal surgery would cause less EG damage. However, on the other hand, pneumoperitoneum applied in minimally invasive surgeries is known to induce I/R injury with reactive oxygen species formation^19^, which is another source of EG damage in addition to inflammatory response. Ultimately, our study revealed that there is a significant EG degradation in patients after minimally invasive laparoscopic or robotic gastrectomy.

The EG is composed of proteoglycans, glycoproteins, glycosaminoglycans, and associated plasma proteins, including albumin^1,2^. Proteoglycans consist of a core membrane-bound protein, such as those of the syndecan family, to which glycosaminoglycan side chains are attached^1,2^. Among glycosaminoglycans, heparan sulfate is the most prominent glycosaminoglycan^1^. Therefore, syndecan-1 (core protein) and heparan sulfate (glycosaminoglycan) have been the most commonly used markers of EG damage in medical and surgical patients^4–13^. The core protein syndecan-1 strongly binds to the cell membrane via a membrane-spanning domain, whereas heparan sulfate is a side chain attached to it^1,2^. Therefore, heparan sulfate is shed first and syndecan-1 is shed later, when severe EG damage occurs^1,3^. In cardiac surgery with cardiopulmonary bypass (CPB), syndecan-1 blood levels elevated following the elevation of heparan sulfate; syndecan-1 levels remained elevated until POD 3, whereas heparan sulfate levels were restored to baseline after weaning from CPB^13^. Therefore, syndecan-1 may be a better marker to detect severe injury and more stable than heparan sulfate.

There was approximately a 20% increase in serum syndecan-1 at POD 1 compared to preoperative values in our patients. This increase is lower than previously reported increases of syndecan-1 of 40–70% at 24 h following major abdominal surgery^5,7^. The discrepancy may arise from the difference in the extent of surgical trauma and the duration of the operation because (1) we studied only minimally invasive surgery, whereas previous studies included open surgery mostly, and (2) we included only gastrectomy, whereas previous studies included various types of abdominal surgery^5,7^. In fact, different types of surgery have been associated with perioperative variations in serum syndecan-1 levels; increases of 30–40% have been reported after lung resection surgery^9,11^, whereas up to 65-fold increases have been reported after major vascular surgery with CPB^4^.

After acute degradation of the EG, 5–7 days are required to endogenously restore the EG thickness in mice^20^. There are no clinically available pharmacologic agents for EG restoration; hence, efforts to minimize EG degradation may be important^3^. Strategies to minimize EG damage include avoiding hypervolemia, supplementing with albumin, and administering pharmacologic agents (e.g., glucocorticoids, antioxidants, and antithrombin III)^2,3,12,21^. Volatile anesthetics were also considered as one of the candidates for EG protection because of their protective effects against I/R injury^14–16^. Sevoflurane has been proven to reduce EG shedding caused by I/R injury through attenuation of the post-ischemic release of lysosomal protease cathepsin B^14^, and through the inhibition of post-ischemic leukocyte and platelet adhesion^15^. Moreover, sevoflurane significantly reduced heparan sulfate shedding after I/R injury compared to propofol by reducing the generation of unmeasured anions^16^. However, contradictory to the reported protective effects of sevoflurane on EG damage in animal models^14–16^, sevoflurane was not superior to propofol in reducing EG degradation in surgical patients^9,10^. In lung resection surgery with one lung ventilation, no difference was observed in heparan sulfate and syndecan-1 concentrations between sevoflurane and propofol groups during surgery^9^. Likewise, no difference existed in heparan sulfate and syndecan-1 concentrations between the two groups up to 90 min after tourniquet release in knee-ligament surgery^10^. These two studies presented design differences compared to our study: sevoflurane or propofol alone was used for general anesthesia maintenance without concomitant use of opioids^9^, and sevoflurane or propofol was used for hypnosis under spinal anesthesia^10^. On the contrary, we administered remifentanil in addition to sevoflurane or propofol because opioids are frequently used during general anesthesia with hypnotic agents. Hence, our study may reflect common clinical settings, and in these conditions we showed no superior effects of volatile anesthesia with sevoflurane/remifentanil over TIVA with propofol/remifentanil on perioperative EG damage.

Although there was no difference in syndecan-1 levels at POD 1 between the groups, the TIVA group showed significantly lower levels of syndecan-1 at the end of the operation compared to the Volatile group (Bonferroni corrected *P* = 0.021). This result is comparable to the significantly lower WBC count and NLR in the TIVA group at the end of the operation compared to the Volatile group (Bonferroni corrected *P* = 0.049 and 0.013, respectively). The NLR is the ratio of neutrophil count to lymphocyte count and is considered as a more sensitive marker of systemic inflammation than the measures of either cell type alone^22^. Therefore, lower levels of WBC count and NLR may reflect an attenuated inflammatory response in the TIVA group, which could have prevented EG degradation during the surgery. However, since sevoflurane also has anti-inflammatory properties^23^ and since CRP levels were similar between the groups, further studies are needed to draw definitive conclusions. Moreover, the protective effects of TIVA against EG damage were only limited to the surgery and were not maintained in the postoperative period. Hence, volatile anesthesia and TIVA can be considered comparable regarding EG protection in minimally invasive abdominal surgery.

This study has some limitations. First, the sample size was not calculated beforehand due to the lack of previous reports. However, the number of patients in our study is larger than that of previous clinical studies comparing syndecan-1 between two treatment groups^7,9,10,12,21^. Second, we evaluated syndecan-1 only until POD 1 based on previous studies^7,12^. However, we evaluated WBC count, NLR, and CRP levels until POD 5 and no differences were found between the groups. Therefore, there may be no difference in syndecan-1 levels between the groups after POD 1 either. Moreover, syndecan-1 returned to baseline levels 48 h after abdominal surgery^5^; thus, evaluation after POD 1 may be meaningless.

In conclusion, EG damage was observed following minimally invasive laparoscopic or robotic gastrectomy with approximately 20% postoperative increase in serum syndecan-1. Although TIVA with propofol/remifentanil showed protective effects against EG damage during the surgery in contrast to volatile anesthesia with sevoflurane/remifentanil, both types of anesthetics could not prevent postoperative syndecan-1 shedding. These results argue against a favorable effect of volatile anesthetics over TIVA on perioperative EG damage in experimental studies, whereas support the previous clinical studies demonstrating comparable effects of these agents on EG damage in surgical patients.

## Methods

### Patients

This study was approved by the Institutional Review Board (IRB) and Hospital Research Ethics Committee (Yonsei University Health System, Seoul, Korea; IRB protocol No. 4-2019-0924) and was registered at http://clinicaltrials.gov (ClinicalTrial.gov, NCT04183296, 03/12/2019). This study was performed in accordance with relevant guidelines and regulations. After obtaining written informed consent from each patient, a total of 136 patients with gastric cancer who were waiting for a laparoscopic or robotic gastrectomy were enrolled between November 2019 and July 2020. Patients were excluded if they fulfilled any of the following criteria: emergency operation; severe hepatic dysfunction (alanine aminotransferase > 3 times of upper normal limit) or end-stage renal disease; allergy or hypersensitivity to sevoflurane or propofol; neurological or psychiatric impairment; history of thromboembolism; and treatment with oral contraceptives or anticoagulants.

### Study design

Patients were randomly assigned to either the Volatile (n = 68) or the TIVA (n = 68) group with the permuted block size of four. In the Volatile group, a bolus of propofol (1.5–2 mg/kg) and target-controlled infusion (TCI) of remifentanil (effect-site concentration [Ce] of 4.0 ng/ml) were administered for anesthetic induction. In the TIVA group, anesthetic induction was achieved by TCI of propofol (Ce of 4.0–4.5 μg/mL) and remifentanil (Ce of 4.0 ng/ml). A commercial TIVA pump (Orchestra Base Primea, Fresenius-Vial, Sèvres, France) was used for TCI of remifentanil and propofol. For anesthesia maintenance, age-adjusted minimal alveolar concentration end-tidal sevoflurane of 0.8–1.0 and TCI of remifentanil were used in the Volatile group, while TCI of propofol and remifentanil were used in the TIVA group to target a Patient State Index (PSI) within the range of 25–50.

### Anesthetic management

Upon arrival at the operating room, all required monitoring devices, including electrocardiography, non-invasive blood pressure, peripheral oxygen saturation, PSI using a SedLine electroencephalograph sensor (Masimo Corp., Irvine, CA), and peripheral nerve stimulator, were applied. After the administration 0.1 mg of intravenous glycopyrrolate as a premedication, anesthetic induction was performed as abovementioned. After loss of consciousness, 1.2 mg/kg of rocuronium was administered to facilitate tracheal intubation, and the degree of neuromuscular blockade was maintained, with 0–2 train-of-four as a target, by rocuronium infusion during pneumoperitoneum. Controlled ventilation was performed at a tidal volume of 7–8 mL/kg with 50% oxygen in air to maintain end-tidal carbon dioxide (CO_2_) at 35–40 mmHg, and a positive end-expiratory pressure of 5 cm H_2_O was applied. Radial artery cannulation was performed in all patients. Hypotension (mean arterial pressure [MAP] < 60 mmHg) was controlled with ephedrine in 4-mg increments or with phenylephrine in 20-μg increments. A forced-air warming system was applied to maintain body temperature at 36–37 °C. Pneumoperitoneum was induced by CO_2_ insufflation, and the intra-abdominal pressure was maintained at 12 mmHg. At the beginning of umbilical closure, 1 μg/kg of fentanyl and 0.3 mg of ramosetron were administered for the prevention of postoperative pain and nausea and vomiting, respectively. All patients were provided with an IV patient-controlled analgesia (PCA) device (Accumate 1100; WooYoung Medical, Seoul, Korea), which consisted of fentanyl infused basally at a rate of 8 µg/h with a bolus dose of 8 µg and a lockout time of 10 min. At the end of the operation, residual neuromuscular blockade was reversed with sugammadex 2 mg/kg intravenously.

### Data collection

Hemodynamic variables (MAP and heart rate) were measured at the following operation time points: before induction, at 30, 60, and 90 min of CO_2_ pneumoperitoneum, and at the end of the operation and arterial blood gas analysis was performed at 90 min of CO_2_ pneumoperitoneum. The primary outcome was serum syndecan-1, which was evaluated at 3 time points: pre-operation (baseline), at the end of the operation, and at postoperative day (POD) 1. Extracted blood samples were centrifuged at 5000 rpm for 5 min at 4 °C to obtain serum and were stored in a -80 °C freezer until analyzed. The analysis was performed by using a specific immunoassay kit (Abnova,Cat. No. KA3851,Taiwan) and all samples were tested in duplicate. In addition, the inflammatory markers, white blood cell (WBC) count, neutrophil-to-lymphocyte ratio (NLR), and C-reactive protein (CRP) were measured at pre-operation, at the end of the operation, and at POD 1, 2, 3, and 5. Postoperatively administered doses of fentanyl at postoperative 6, 12, and 24 h were registered using the PCA device, which automatically recorded the delivered quantities of drug every 30 min. The number of patients who required rescue analgesics (25 mg of intravenous pethidine) was also registered up to postoperative 24 h.

### Statistical analysis

The primary outcome was perioperative syndecan-1, which was compared between volatile anesthesia and TIVA groups. Previous studies on perioperative changes of syndecan-1 concentration after abdominal surgery with pneumoperitoneum were not found; thus, this study was designed with 68 patients per group. This number is higher than that of previous studies on the effect of sevoflurane and propofol on perioperative syndecan-1 shedding^9,10^.

Continuous variables are shown as mean ± SD and categorical variables are expressed as number of patients (percentage). Group differences for continuous variables were analyzed by the Student’s *t* test, which is valid according to the central limit theorem because of the large number of patients (65 and 67). Differences in categorical variables were checked by the Chi-square test or by the Fisher’s exact test: the former was employed if the portion of cells with an expected cell frequency of less than 5 was less than 20% of all the cells, and the latter was employed otherwise. For repeated-measure variables, a linear mixed model analysis was employed to determine group and time effects and a compound symmetry covariance structure was used for the within-subject effect. The Bonferroni correction was used to adjust for multiple comparison in post-hoc analyses. A *P* value of less than 0.05 was considered to be significant. All analyses were conducted using SAS version 9.4 (Cary, NC).
